# Assessment of Mood and Hope in Critically-Ill Patients as a Basis for the Improvement for the Palliative and Hospice Care

**DOI:** 10.3390/ijerph19169987

**Published:** 2022-08-13

**Authors:** Bożena Baczewska, Bogusław Block, Mariola Janiszewska, Krzysztof Leśniewski, Agnieszka Zwolak

**Affiliations:** 1 Department of Internal Medicine in Nursing, Medical University of Lublin, Chodźki 7 Street, 20-093 Lublin, Poland; 2 Institute of Psychology, The John Paul II Catholic University of Lublin, Al. Racławickie 14, 20-950 Lublin, Poland; 3 Department of Medical Informatics and Statistics with e-Health Lab, Medical University of Lublin, Jaczewskiego 4 Street, 20-090 Lublin, Poland; 4 Department of Orthodox Theology, The John Paul II Catholic University of Lublin, Al. Racławickie 14, 20-950 Lublin, Poland

**Keywords:** basic mood, hope, terminally ill patients, cancer, palliative care

## Abstract

The aim of the research is to present a level of hope in people in the terminal phase of cancer who differ in terms of mood: cheerful vs. sad. The study group consisted of 246 patients. Their average age was 59.5. The youngest respondent was 18 and the oldest was 90. The Personal Card tests by T. Witkowski (KI) and B.L. Block (NCN-36), designed for people struggling with serious life-threatening diseases, were used. The test consists of four scales distinguished by factor analysis. Each scale comprises of eight items. The following are used to study hope: the situational dimension—health; the telek-temporal dimension—goals; the spiritual dimension—religious beliefs; and the emotional-affective dimension—motivations. In the global view, the hope of the subjects was moderate. In the situational dimension—health, the telek-temporal dimension—goals, and the spiritual dimension—religious beliefs, people with a generally cheerful mood had stronger hope, in comparison to people with a sad mood. Only in the emotional-affective dimension—motivations, did people with a sad mood manifest stronger hope in comparison to people with a cheerful mood. The conducted research allowed us to conclude that mood is one of the determinants of hope in terminally ill cancer patients.

## 1. Introduction

Accompanying a patient in the terminal phase of a cancer disease in the last months, weeks, days, or hours of his/her life is a special time that presents an opportunity to experience closeness. This is a time of care in an atmosphere of respect, gentleness, and understanding that gives hope to the dying person that he/she will not be alone in the face of death, and that all activities will be carried out with the dignity that every person deserves, that everything will be done to ensure the highest quality of life in all phases of dying.

There is no clear definition of hope in the psychological literature. Hope is sometimes perceived as an emotion [1] or “emotional trust or an optimistic way of thinking (…) which helps in achieving a high quality of life” [2]. Some scholars describe it as a psychological mechanism [3] or a thought process [4]. Hope is also sometimes analysed in the context of an attitude contrary to depression, despair, or pessimism [5]. Indeed, as early as the 1960s, Beck linked the symptoms of depression with the loss of hope in the context of health and disease. He created the concept of depression due to the loss of hope. To this end, he also developed a depression measurement tool called the “Beck Depression Inventory” [6,7,8]. Similar studies were also carried out in later years, including by Breitbart et al. [9], Cheavens [10], and Fehring et al. [11].

According to Erik Erikson [12,13], hope is the first and most essential virtue closely related to a person’s life situation. He emphasised the importance of the development of hope during infancy as crucial for shaping the entire structure of personality, which is the effect of positively solved psychosocial crises in the course of human life.

Many authors note that hope is an inseparable element of human life [14], of the “human condition” [15,16,17,18] and even “a specifically human experience” [19].

In the field of positive psychology, Snyder regards hope as a dynamic and multidimensional thought process consisting of three integrally connected elements: goal, willpower, and waypower [14]. All three components are necessary and affect the quality of hope. Having hope, in Snyder’s theory, means having great mental strength, the ability to plan and create new solutions (waypower), and the ability to define tasks and goals [14,20,21].

Mood is defined as the affective state having valence, i.e., value (positive or negative) and intensity (usually low), also containing more or less precise expectations as to feelings that will occur in the near future [22]. It is a persistent emotional tinge of all experiences that are subjectively experienced and reported but noticeable by the environment. Moreover, it is a component of a temperament, and it exhibits constant and individualised features. In the varying conditions of human life, there are natural short-term mood changes, subject to a given situation.

According to Thaler’s concept, mood is related to the general stimulation of the organism, which consists of two subjectively felt phenomena: energy- and tension-related. The energy dimension presents two opposite poles, i.e., energy and fatigue. The tension-related factor is between the stress and calm poles. Both dimensions are separate, but changes in one may trigger changes in the other [23].

Usually, however, optimal mood states are associated with a feeling of low tension and high energy. The most negative states are generally associated with a sensation of relatively low energy and high tension. Self-regulation of mood is associated with the manifestation of actions aimed at increasing energy, reducing tension, or triggering both changes simultaneously.

There are many typologies of mood in the literature on the subject. For the purposes of this work, the typology proposed by Witkowski [24] was adopted. This is characterised by the simplicity of the terms used and enables easy self-assessment for patients.

The main goal of the research is to show hope to people in the terminal phase of cancer, which differs in terms of their basic mood.

## 2. Materials and Methods

In the present study, we followed the methods of Baczewska et al., 2019a, Baczewska et al. 2019b, Baczewska et al., 2020a, and Baczewska et al., 2020b [25,26,27,28]. It involved 246 participants at the terminal phase of the neoplastic disease. The patients were in day care, 24/7 palliative and hospice care units in 17 centres in Poland.

The criterion for selecting patients for the study was the patient’s consent, a previously diagnosed cancer in the terminal phase, aged from 18 years, and the patient staying in a palliative or hospice care unit. 

When selecting participants for the study, it was also taken into account that in the terminal stage of cancer, patients often have limited possibilities for cognitive functioning. The reasons for this are wasting of the body (cachexia-anorexia syndrome) and tumor weakness syndrome (asthenia), as well as the influence of strong medications. Patients with limited cognitive functions were excluded from the study. The condition of the potential participants was established by the highly qualified nurses who directly looked after them. These nurses specialised in palliative care and had extensive experience of working with patients in the terminal stage of cancer. They, therefore, assessed the patient’s functional capacity in the cognitive sphere and their ability to think logically.

Overall, most patients were willing to participate in the study, but some refused due to malaise. Not all consenting patients managed to complete the study. Still, less than 10% of all participants failed to complete the study because of too much fatigue or sudden health breakdown and death.

Before starting the survey questionnaire, consent was obtained from the patient, and they were informed about the anonymity of the study. The study was conducted by filling in questionnaires, but, additionally, therapeutic activities were included. Due to the poor health of the study participants, the questionnaire was completed by the person conducting the study, and the time of the study was adjusted to the abilities and needs of each patient. During the interview, only the patient and the person conducting the survey were in the room. Apart from collecting material for research, the aim was to provide psychological support to patients. More than once, the interview with the patient turned into a therapeutic conversation. 

For the research, the following tools were used: a Personal Card (KI) constructed by T. Witkowski and the Block Hope Scale (NCN-36).

A Personal Card (KI) is a set of data selected from a large individual sheet by Witkowski [24] and supplemented with new data, adapted to the subject of new research. The Personal Card for people in the terminal phase of neoplastic disease contained data for the description of the group, enabling them to be evaluated as variables for further analysis in terms of their possible relationship with NCN-36 results. The basic goal of constructing the Personal Card was to collect systematised information about the person in the terminal phase of cancer disease and their environment. The intention being that in conducting collective research via interviews, it was important that the tool be simple and the information easily accessible.

In addition to basic information such as age, gender, place of residence, marital status, education, and the time elapsed since becoming ill, variables concerning the emotional-motivational sphere such as general mood, general peace, and ease of revealing feelings were introduced. As regards the general mood distinguished by Witkowski [24], two types of mood were distinguished: cheerful and sad. In addition, the following subtypes were identified: cheerful unstable, cheerful stable, sad unstable, and sad stable. This division was adopted due to the ease of mood self-assessment.

The Block Hope Scale (NCN-36) has been developed for the study of people struggling with severe, life-threatening diseases. It consists of 36 items. The respondent answers the seven-level Likert Scale. The test consists of 4 scales distinguished in the factor analysis. Each scale consists of 8 items. There were also 4 buffer questions included in the test.

The test allows assessment of hope in the situational dimension-health, the telek-temporal dimension-goals, in the spiritual dimension-religious beliefs, and in the emotional-affective dimension-motivations.

The situational dimension is associated with the uncertainty of waiting for recovery and/or health improvement as well as concerns about the effectiveness of treatment and is associated with the level of confidence in the professionalism of the doctor and the belief in the effectiveness of medicine (scientific and unconventional). This dimension of hope seems to correspond with the telek-temporal dimension (the object of hope), but it is a separate factor.

The telek-temporal dimension-relates to hope, which is connected with the patient’s attitude to their future. Patients see many reasons to live because they are convinced that there will be more interesting things to experience, or they have important goals to achieve, unfulfilled dreams, plans, and desires. This component of hope is indirectly linked to the motivational function of hope, which prompts the sick to take action and try to regain health. It makes them care about the progress of treatment, motivates better cooperation with the doctor, and compels more positive adherence to medical recommendations.

Spiritual-religious hope has reference to a ‘Higher Being’, a personal ‘God’ in whom they trust and in whom they hope. Those who have achieved high scores in this subscale will be willing to entrust their lives and their future to ‘God’, trusting in ‘His’ mercy and protection, which will give them spiritual support and a sense of peace in the difficult experience of illness. For a person who has such a hope, there is a perspective of eternal life, which goes beyond the limit of death-into eschatological time. A strong hope in this dimension may favour the acceptance of their life situation but may also trigger passivity and apathy.

The affective component of hope means that one does not give in to fear, and one’s attitude to illness is characterised by bravery and courage. Moreover, when thinking about an uncertain future, one feels inner peace. This component of hope seems to counteract the feelings of sadness or depression and tension resulting from a difficult situation and an uncertain future. Results in the affective dimension indicate the possibility of experiencing ambivalent feelings by people who are sick with the fear of an uncertain tomorrow, suffering and death, and, at the same time, optimism and courage.

The test obtained a high reliability measured by the Cronbach’s alpha coefficient, which was 0.92. In particular, on scales, it was within the limits of 0.72–0.86, which indicates the internal coherence of the tool. The range of results is from 1 to 7. Interpretation is based on averaged results. Very strong hope is evidenced by results from 6.0–7.0, strong 5.0–5.99, moderate 4.0–4.99, weak 3.0–3.99, very poor 2.0–2.99, and hopeless-1.0–1.99. The strength of hope felt by the respondent reflects the global result. The patient’s hope profile creates results on each of the four scales.

### Statistical Analysis

In order to present the results of our own research, basic descriptive statistics were applied. These are the arithmetic mean (M), standard deviation (SD), as well as quantitative and percentage distribution of results. In contrast, statistical analysis of differences was based on the Student’s t-test. A minimum level of statistical significance (*p*) of at least 0.05, a significance coefficient (t), and degrees of freedom (df) were adopted.

## 3. Results

The study involved 246 participants at the terminal phase of the neoplastic disease. The subjects were 18 to 90 years old, and the average age for the study group was 59.5. The largest group of 99 people were aged 51–65 (40.24%); 14 were aged up to 35 (5.69%); 86 people were over 65 years (34.96%) and 47 were aged from 36 to 50 years (19.11%). The majority of respondents were women—150 (60.98%) and urban residents—192 (78.05%). Furthermore, the majority of participants were married—117 (47.56%). The second largest group were widowed—67 (27.24%) and the third largest group were divorced—32 (13.01%). The least numerous group were single—30 (12.2%). A third of respondents had completed secondary education—82 people (33.33%). Two equally numerous groups of patients with basic and vocational education participated in the study—59 people (24.22%). Respondents with higher education comprised 29 people (11.79%) and 17 (6.91%) had an incomplete higher education. The results of the research concerning the hope of people being given palliative and hospice care depending on mood are presented in Table 1.

As presented in Table 1, stronger hope is revealed by people with a cheerful mood in global terms (Global Score = 4.76) than in people with a generally sad mood in global terms (Global Score = 4.34). This was confirmed at a very high level of statistical significance (*p* < 0.0000). A similar result may be observed in the NCN-36 sub-scales, i.e., dimensions of hope. With regard to the situational dimension of hope (health), people in a cheerful mood exhibited stronger hope than people in a sad mood (*p* < 0.0001). In addition, people in a cheerful mood have stronger hope for positive events in the future (the telek-temporal dimension of hope) than people in a sad mood (*p* < 0.0001). Greater trust in ‘God’, a ‘Higher Being’, and placing hope in ‘Him’ (the dimension of values and convictions of personal hopes) are also characteristic of positively cheerful people than those with a sad mood (*p* < 0.0001). However, feeling inner peace and not giving in to fear (emotional and motivational dimension of hope) is stronger in people with a sad mood as compared to people with a cheerful mood (*p* = 0.02).

The distribution of hope results in the researched groups, is presented in Table 2. It shows the sources of differences between global mean averages for hope among the respondents.

In the two intervals of low and very low scores, that is, from the lack of hope to poor hope, there was no person (0.0%) in the group of people in a cheerful mood, whereas among the sad, there were eight people (5.7% of the respondents). In three middle intervals close to moderate hope, 97.2% of people have a cheerful mood and 92.14% of people have a sad mood. On the other hand, in the intervals of very strong hope, there were 2.8% of cheerful people and 2.1% of sad people. In the group of people with a sad mood, there is a mild upward trend in the number from the first interval (lack of hope), *f* = 1.

A similar result in people with a sad and cheerful mood is found in reference to moderate hope, but here there are more people with a cheerful mood. In the intervals from moderate hope to very strong hope, similar differences in people with cheerful and sad moods may be observed.

As displayed in Table 3, in the group of respondents with a sad mood, 61.4% of all people were below the theoretical average, and the distribution of results shifted towards low results. These results indicate a weak hope in the situational dimension (health) in six out of ten people with terminal phase cancer. In contrast, 47.2% of all people with a cheerful mood have poor hope in this dimension.

It is also worth paying attention to the number of people with extreme results in terms of hope in the health dimension. Among sad people, 10.7% indicated low results for hope in the health dimension, and almost 25% of all people have very poor hope in the health dimension. In contrast, 7.2% of sad people and 11.3% of cheerful people expect recovery. 

As to hope expressed as the expectation of the fulfilment of dreams and desires or the implementation of certain plans in the future, in the “Goals” subscale, the results are also differently distributed among sad and cheerful people. Low scores were obtained by 30% of sad and 9.4% of cheerful people. However, 12.1% of sad people and 23.6% of cheerful people were almost certain that their dreams would be fulfilled in the future.

Faith and the system of beliefs are an important element of hope in the terminal phase of the disease, that is, in situations where nothing or little depends on us. In this respect, the hope of respondents is shifting towards high results in both groups. There are as many as 96.2% of all respondents with a cheerful mood and 82.1% with a sad mood. This spiritual dimension of hope turns out to be the main aspect of hope for respondents.

Results in the dimension of the motivational sphere of hope are distributed in a different way. They are the most similar to the normal curve, where the vast majority of results are near the average. Thus, 87.9% of sad and 92.5% of cheerful people found themselves in the two middle intervals. This dimension of hope seems to be the most basic structural element that would belong to the permanent personality structure and is least dependent on mood or the situational factor, which is a serious disease with an unfavourable prognosis.

## 4. Discussion

The results presented in this paper are part of comprehensive studies concerning the multifaceted assessment of hope among terminally-ill cancer patients undergoing palliative and hospice care in Poland and are a continuation of the studies published by Baczewska et al. [25,26,27,28]. In the present paper, the problem of the level of hope, considered in the four dimensions included in the applied NCN-36 test, was analysed in the aspect of the patient’s mood.

As found in the present study, people with a cheerful mood have a statistically significantly stronger hope in the situational dimension-health (*p* = 0.0027), in the telek-temporal dimension-goals (*p* < 0.0001) and in the spiritual dimension-religious beliefs (<0.0001), and a statistically significantly weaker hope in the emotional-affective dimension motivations (*p* = 0.0204) in comparison to people with a sad mood. Patients with a cheerful mood are also characterised by much higher hopes in global terms (*p* < 0.0001). The lack of hope and weak hope are manifested only by people with a sad mood—5.71% of all patients with a sad mood have such hope, whereas mood does not differentiate the group of patients with moderate hope or with very strong hope. Regardless of mood, in the structure of hope of people dying of cancer, the spiritual dimension of hope dominates, and its basic elements are inner peace and trusting hope (emotional and motivational dimensions of hope). The conducted research allowed us to conclude that mood is one of the determinants of hope in terminally ill cancer patients.

Other researchers are also interested in the issues of hope and mood in terminally ill cancer patients. They show the relationship between patients’ mood and quality of life and point towards the need for psychological support of dying people, noting at the same time that the dying process is not always related to experiencing depression.

Such studies were, inter alia, conducted by Łopuszańska et al., who, in assessing patients with lung cancer, reported depression symptoms of varying intensities in 32.25% of them. In 25.8%—it was mild, 6.45%—moderate, and 4.83%—severe. The results indicate that the severity of depression increases with decreasing general physical functioning and is not related to the severity of pain [29]. The results of our own research are in line with that of Rabkin et al. They showed that patients approaching the end of their lives experienced both positive and negative moods. Rabkin et al. noted that during the last visit before their deaths, a significant minority of respondents met the criteria of depression. Still, a surprising discovery was that deep depression is not an integral part of the process of dying in the terminal phase of cancer. Therefore, the authors of this report stress the need to verify the classification of sadness, loss of interest, and thoughts that “it would be better to die” in the last days of life as psychopathology [30].

Utne et al. conducted research on the relationship between mood disorders and pain, hope, and quality of life in a group of patients with cancer pain who received regular opioid analgesia. They found more frequent anxiety and depression in younger patients and in women. Only minimal differences in the severity of pain were found in the groups of patients with anxiety, depression, and those suffering from both disorders at the same time. In contrast, a group of patients aged four to twelve, in which neither anxiety nor depression was diagnosed, had a lower level of pain interaction than in the other three groups. In addition, patients with both mood disorders reported a significantly worse quality of life. The group of patients experiencing both anxiety and depression in these studies was numerous and accounted for 44% of all respondents [31]. Our studies also found that mood influences the quality of life of patients, as only sad patients experienced low hope or loss of hope. In these study participants, hope was significantly lower in terms of health, goals, and religious beliefs, and in general terms, than in patients with a cheerful mood.

The authors of other studies, like us, also point to the need to improve the care of patients with terminal cancer, and in particular, to provide individualised care for each person. Some studies indicate deficiencies in the current psychological care for dying patients. This allows us to state that the improvement of this care should be an ongoing subject of research.

Acquaye found that patients with relapse had a higher overall mood disorder compared with those without relapse. Patients with a higher level of hope also reported lower mood disorders. Patients with relapsed cancer reported lower hope and worse mood disorders than those who were newly diagnosed or without relapse. The authors of the publication emphasise the need to adapt interventions specifically to the individual’s needs in terms of improving the quality of life during the entire course of the disease. Interventions should include measures aimed at facilitating positive coping strategies [32]. Similarly, our study indicated a higher level of hope in patients with a cheerful mood in almost all dimensions except the affective.

In research concerning the psychological aspects of depression in cancer patients, Die Trill states that depression is common in cancer illness and should be diagnosed correctly. Treatment of depression should not only focus on the patient but should also include family members and oncological staff [33].

Our own research shows that sad people on the verge of losing hope in the health dimension represent 10.7% of all patients. However, almost 25% of all terminally ill individuals have very poor hope in this dimension. These people need to be especially protected against depression, which is the result of losing hope. The latest literature emphasises the need not only to provide support to people in the terminal stage of cancer but also to adapt this support to the current needs of patients and their caregivers. According to a study by Graham-Wisener et al., only a small number of patients with oesophageal cancer and their caretakers benefit from psychological support, and the support they receive is not adequate for their needs [34].

Jabbour et al. focused on the unmet needs of patients with head and neck cancer in relation to the provision of support. About 50% of all respondents reported that they did not receive any information about coping with stress and anxiety. Still, most patients received minimal information on the availability of support groups for patients (56%) [35].

In turn, Li et al. emphasise that cancer patients need not only advanced therapy but also spiritual counselling. This is because psychological support and intervention can reduce the negative experiences of patients with cancer and improve the quality of their lives [36]. Another important issue is the aspect of outpatient support. Unfortunately, while Buchhold et al. indicate that psychosocial services for hospitalised patients have been extended in recent years, the structure of outpatient care in terms of psycho-oncological support is far from satisfactory [37].

### Clinical Implications

The aim of our research was to show that hope in terminally ill cancer patients depends on their basic mood. This may help to improve the quality of patient care in the terminal phase of cancer. By learning about the problems and needs of terminally ill cancer patients, it is easier to adjust the care medical staff give them. The results of the research may allow individualisation of care for dying patients to improve their situation in the last stage of their life so that they can die with dignity.

Special attention should be paid to patients with very strong and very weak hope. Very strong hope occurs with a similar frequency among patients with cheerful and sad moods. These are people who hope for a miraculous recovery or have such a hope that allows them to live in hope despite what they hear. These are probably people who do not want to know that theirs is an unfavourable prognosis, and they completely disagree with their situation. They will use defensive mechanisms of denial and displacement (which probably allow them to continue to survive). They want to live so much that they do not give in to illness. They fight to the end, and they cannot come to terms with the inevitable; the perspective of imminent death. It may be said that they are in the phase of illusional hope, which, however, allows them to cope with their fate [20,38].

Very weak hope or lack of hope, on the other hand, appears only in patients with a sad mood. These patients have to be protected against depression, which is the result of losing hope [6,7,8,9,10,11]. Both patients with very strong and very weak hope are a challenge for palliative and hospice care staff. In this situation, helping becomes the art of solving a dilemma, how to help, not to harm while conducting conversations aimed at making the hope realistic.

## 5. Conclusions

Based on the research carried out, the following conclusions can be drawn: (1) mood seems to be one of the determinants of hope in terminally-ill cancer patients; (2) terminally-ill cancer patients with a cheerful mood have a higher level of hope in general terms, as well as in situational, telic-temporal and spiritual dimensions; (3) terminally-ill cancer patients with a sad mood have a higher level of hope in emotional-affective dimension; (4) lack of hope or low hope only arises in the terminally-ill cancer patients with a sad mood; (5) moderate and strong hope are observed with a similar frequency in patients with a happy and sad mood.

## Figures and Tables

**Table 1:** Hope of people in the terminal phase of cancer due to mood in the study group *N* = 246.

NCN-36	Cheerful *N* = 106		Sad *N* = 140		Difference *M*	*t*	*df*	*p*
	*M*	*SD*	*M*	*SD*				
**Global Score**	4.76	0.66	4.34	0.82	0.42	4.37	244	<0.0001
**Health**	4.15	1.29	3.63	1.38	0.52	3.03	244	0.0027
**Goals**	5.13	0.99	4.46	1.26	0.67	4.53	244	<0.0001
**Religious Beliefs**	5.73	0.93	5.04	1.23	0.71	4.82	244	<0.0001
**Motivations**	4.04	0.57	4.23	0.64	−0.19	−2.33	244	0.0204

**Table 2:** Distribution of the percentages of global results for hope in people in the terminal phase of cancer of the studied group *N* = 246.

Class Intervals for the Global Score of NCN-36	Cheerful (*N* = 106)		Sad (*N* = 140)	
	Frequency (f)	%	Frequency (f)	%
1.00–1.99	0	0.00	1	0.71
2.00–2.99	0	0.00	7	5.00
3.00–3.99	10	9.43	35	25.00
4.00–4.99	57	53.77	68	48.57
5.00–5.99	36	33.96	26	18.57
6.00–7.00	3	2.83	3	2.14
Total	106	100.00	140	100.00

**Table 3:** Distribution of the results in the NCN-36 subscales due to the mood of the respondents.

Subscales Group		Health		Goals		Religious Beliefs		Motivations	
		*f*	*%*	*f*	*%*	*f*	*%*	*F*	*%*
**Sad**	1.00–1.99	15	10.72	5	3.57	2	1.43	0	0.00
	2.00–2.99	34	24.29	15	10.71	9	6.43	3	2.14
	3.00–3.99	37	26.43	22	15.71	14	10.00	39	27.86
	4.00–4.99	28	20.00	51	36.43	33	23.57	84	60.00
	5.00–5.99	16	11.43	30	21.43	47	33.57	12	8.57
	6.00–7.00	10	7.14	17	12.14	35	25.00	2	1.43
**Total**		140	100.00	140.00	100.00	140	100.00	140	100.00
**Cheerful**	1.00–1.99	2	1.89	0	0.00	0	0.00	0	0.00
	2.00–2.99	18	16.98	1	0.94	1	0.94	2	1.89
	3.00–3.99	30	28.30	9	8.49	3	2.83	48	45.28
	4.00–4.99	22	20.75	38	35.85	15	14.15	50	47.17
	5.00–5.99	22	20.75	33	31.13	38	35.85	6	5.66
	6.00–7.00	12	11.32	25	23.58	49	46.23	0	0.00
**Total**		106	100.00	106	100.00	106	100.00	106	100.00

## Data Availability

The data presented in this study are available on request from the corresponding author. The data are not publicly available due to privacy restrictions.
